# The Decision to Wear a Face Mask as a Protective Behavioral Measure Against COVID-19: Survey Results From Greater Kampala Metropolitan Area, Uganda

**DOI:** 10.3389/fpubh.2021.675734

**Published:** 2021-10-21

**Authors:** Paul M. Bukuluki, Peter Kisaakye

**Affiliations:** ^1^Department of Social Work and Social Administration, Makerere University, Kampala, Uganda; ^2^Department of Population Studies, Makerere University, Kampala, Uganda

**Keywords:** COVID-19, face masks, greater Kampala metropolitan area, Uganda perspectives, Uganda

## Abstract

The use of face masks is one of the behavioral measures used to prevent COVID-19 infection. Despite the positive contribution of face masks, there is uncertainty surrounding face mask wearing in low-income countries. Using data from 1,054 respondents in Greater Kampala Metropolitan area, we investigate the variation in face mask wearing inside and outside public spaces. Results indicate that more than three quarters of the respondents wore a face mask always outside public spaces and slightly more than half wore a face mask sometimes inside public spaces. Irrespective of location (inside or outside public spaces), respondents were more likely to wear facemasks sometimes or always to prevent COVID-19 infection. There is need to raise awareness about face mask wearing and its efficacy to prevent COVID-19 infection.

## Introduction

The emergence of COVID-19 in December 2019 (1), called for protective measures to curb its spread (2). COVID-19 is a respiratory disease that is caused by acute respiratory infection (3). Other than social distancing (4), lockdown (5), handwashing (6, 7), the use of face masks is one of the behavioral measures used to prevent the spread of COVID-19 (3, 8–11). The use of face masks falls under a low-cost non-pharmaceutical intervention (12).

In low income countries (LICs) such as Uganda, the use of face masks can be an important and low cost preventive measure against cross-contamination among medical personnel, patients and health care workers (3). Face masks act as a barrier that can prevent one from inhaling infected viral particles through the mouth or nose (13–16), hence also prevent the development of respiratory problems such as breathing difficulty, disease (9).

Mass wearing of face masks can also lead to a reduction in community infections (17–20). While wearing of face masks is affordable and effective against the spread of COVID-19, available evidence in low-income countries points to uncertainty surrounding the quality of face masks, poor use, shortage, and efficiency (21–24). The effectiveness of wearing a face mask can also be affected by the way people wear, remove and dispose them (17).

Moreover, some people just choose not to wear face masks despite having knowledge about the spread of COVID-19 and the effectiveness of using face masks (25). For example, the use of face masks was found to be low among Nigerians despite having knowledge about the spread of COVID-19 (26). In Sudan, only a third of residents were wearing face masks (27). In Ethiopia, half of 331 respondents reported not to have worn a face mask before leaving home when they were going to a crowded place (28). A recent study in Uganda revealed that having knowledge about the use of face masks is not universal—with only 68% having received information about the use of face masks (29). Reasons for non-use of face masks include cost (25), poor education about face mask use (30–32), the perception that people cannot be infected with COVID-19 (33) or thinking that the spread of COVID-19 is through other means such as mosquito bites (34) or meat consumption (35), and stigma attached to those who wear masks (36).

The World Health Organization (WHO) recommends the use face masks particularly by infected and health professionals while the US Centre for Disease Control and Prevention (CDC) recommends that for effective prevention of transmission, everyone should wear a mask (17, 21, 37, 38), there is increasing evidence that the use of face masks prevents COVID-19 transmission (10, 20).

This study examines the factors that influence the decision to wear or not wear a face mask among urban dwellers in Kampala, Uganda. Further, we investigate the variation in the face mask use behavior inside and outside public spaces in Kampala, Uganda as well as the age and sex differences. We focus on Kampala, Uganda because of three reasons. First, greater Kampala, Uganda is a high-risk area for COVID-19 infection given it has the highest number of COVID-19 cases (39). At the time data collection was carried out, Uganda had registered a total of 1,313 cumulative cases of COVID-19 as at 10th August 2020 (40). Second, it embodies urban dynamics such as slums, congestion, traffic jam that provide ground for ease in transmission of COVID-19, unlike other urban areas in Uganda (41, 42), and last, greater Kampala has the highest urban population density that may contribute to congestion and undermine social distancing guidelines (43).

This study informs the design of effective and context sensitive behavioral change communication strategies aimed at promoting use of face masks for prevention of COVID-19 infection. The results in this study can help to shed light on the level of adherence to the recommended practices of wearing face masks during the COVID-19 global pandemic in urban cities in low income countries such as Kampala, Uganda.

## Data and Methods

The study was based on analysis of data collected for a period of 3 months (August–November 2020)—at a time when the lockdown and mobility restrictions were lifted (39, 44). This study was part of the project that aimed to investigate the impact of COVID-19 on Social Support Systems. Using the formula of simple random sampling with proportions, *p* = 0.107, *q* = 0.893, *z* = 2.33, margin of error = ±2 and 95% level of significance, we estimated a sample of 1,300 respondents.

The data analyzed were collected from 1,054 Greater Kampala Metropolitan urban respondents. In this study, greater Kampala includes the area under Kampala Capital City Authority (KCCA) and its surrounding suburbs of Mpigi, Mukono and Wakiso.

Accidental sampling was used to recruit respondents. Accidental sampling is a non-probability sampling method that is used by researchers when they want to take advantage of easy access, geographic proximity, availability and willingness of people to participate in the study (45). This sampling approach was adopted for this study given the prevailing circumstances of COVID-19 (such as minimal movements to people's households, shorter hours of daily work due to curfew) at the time data collection was done. Interviewers (men and women and of different age groups) would position themselves in busy spots. Interviewers would kindly ask people passing by to stop, and only those who were willing and consented to participate in the study would be interviewed. Research assistants were well trained for 3 days to collect data using Computer Assisted personal Interviewing (CAPI) technology. A pre-test was carried out prior to the main data collection exercise and all comments from the pre-test were incorporated into the final revision of the questionnaire.

During the interview process, all Standard Operating Procedures (SOPs) for collecting data during the COVID-19 pandemic as guided by the World Health Organization were followed (46). As part of observing ethics, interviewers insisted that respondents must wear a face mask during the consenting process of the interview. In instances where the respondent did not have a face mask, the interviewer(s) availed a face mask and requested the interviewee to wear it.

We collected information on the age (18 years and above) and sex of the respondents (female or male). We also collected information on the frequency of wearing a face mask inside or outside public spaces. Responses to these questions were “Never,” “Sometimes,” or “Always.” Respondents were asked whether wearing a face mask inside or outside public spaces is a protective measure against COVID-19 infection. In this paper, inside public spaces refers to malls, shopping centers, supermarkets, banks among others while outside public spaces refers to streets, roads, playgrounds among others. This question was used to measure the belief about face mask efficacy (as the outcome variable). A response to this question was either “Yes” or “No.”

We used STATA software version 15.0 (STATA Corporation, College Station, TX, USA) (47) for data analysis to present frequency distributions and bivariate relationships. Ethical considerations required during the data collection process were followed. Permission to conduct the study was granted by the School of Social Sciences Research Ethics Committee at Makerere University (MAKSS REC 09.20.452). We sought consent from all respondents who participated in the study. All respondents who participated in the study provided verbal informed consent. The interview duration ranged from 30 to 45 min.

## Results

### Distribution of Respondents

The response rate for this study was 81% (1,054), given that we had estimated a total sample of 1,300 respondents. Table 1 shows that slightly more than half (53%) of respondents were female and close to half of respondents (49%) were in the age group 25–34 years. The results indicate that respondents who were 45 years and above constituted the least proportion in the sample. The majority of respondents (52%) wore a face mask sometimes inside public spaces while 78% wore a face mask always outside public spaces.

**Table 1:** Distribution of respondents.

**Variable(s)**	**Number**	**Percent**
**Sex of respondent**		
Female	559	53.3
Male	489	46.7
**Age of respondent**		
18–24	209	20.0
25–34	509	48.6
35–44	243	23.2
45+	86	8.2
**Frequency of face mask wearing inside public spaces**		
Never	91	8.6
Sometimes	547	52.3
Always	407	39.0
**Frequency of face mask wearing outside public spaces**		
Never	58	5.5
Sometimes	171	16.3
Always	819	78.2
Total	1,054	100

**Figure 1:**
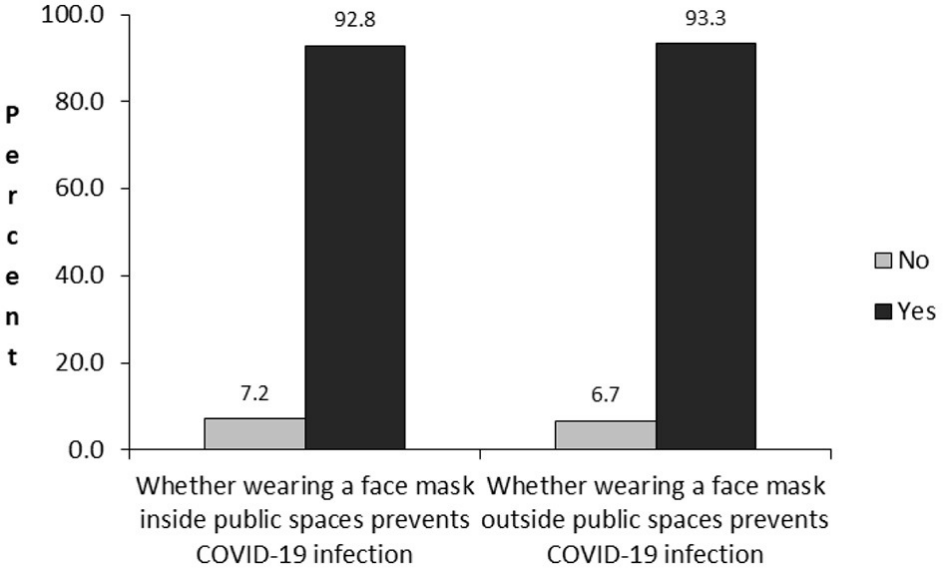
Belief about face mask efficacy.

Respondents were asked whether wearing a face mask inside or outside public spaces can prevent COVID-19 infection. These questions were used to measure the belief about face mask efficacy. Figure 1 shows that the majority of respondents agreed that wearing a mask inside public spaces (92.8%) or outside public spaces (93.3%) can prevent COVID-19 infection. These results indicate that nearly all respondents believe that face masks are effective at preventing COVID-19.

### Relationship Between Selected Variables and Belief About Face Mask Efficacy

Table 2 shows results of bivariate relationships between selected variables and whether wearing a face mask inside public spaces can prevent COVID-19 infection. The results indicate a significant relationship between the frequency of face mask wearing inside or outside public spaces. Overall, other than respondents who never wore a face mask, the majority of respondents agreed that wearing face mask inside public spaces can prevent COVID-19 infection.

**Table 2:** Relationship between selected variables and whether wearing a face mask inside public spaces can prevent COVID-19 infection.

**Variable(s)**	**No (%)**	**Yes (%)**	**Chi-square** **(P-value)**
**Sex of respondent**			0.666 (0.414)
Female	6.5	93.5	
Male	7.8	92.2	
**Age of respondent**			2.793 (0.425)
18–24	9.1	90.9	
25–34	6.3	93.7	
35–44	8.0	92.1	
45+	4.7	95.4	
**Frequency of face mask wearing inside public spaces**			268.0.42 (0.000)*
Never	49.5	50.6	
Sometimes	4.2	95.8	
Always	1.7	98.3	
**Frequency of face mask wearing outside public spaces**			237.493 (0.000)*
Never	56.9	43.1	
Sometimes	10.0	90.0	
Always	3.1	96.9	
Total (%)	7.2	92.8	
Total (N)	75	972	

* *p < 0.05*.

Table 3 indicates that the age of the respondent, frequency of face mask wearing inside or outside public spaces were significantly related to belief in face mask efficacy. Irrespective of age, most respondents agreed that wearing a face mask outside public spaces prevents COVID-19 infection. Other than respondents who never wore a face mask outside public spaces, respondents who sometimes or always wore a face mask agreed that wearing a face mask outside public spaces prevents COVID-19 infection. The results in Table 3 show that irrespective of the frequency of wearing a face mask inside public spaces, the majority of respondents agreed that wearing a face mask outside public spaces prevents COVID-19 infection.

**Table 3:** Relationship between selected variables and whether wearing a face mask outside public spaces prevents COVID-19 infection.

**Variable(s)**	**No (%)**	**Yes (%)**	**Chi-square** **(***P***-value)**
**Sex of respondent**			0.131 (0.717)
Female	6.5	93.5	
Male	7.0	93.0	
**Age of respondent**			7.973 (0.047)*
18–24	11.1	88.9	
25–34	5.9	94.1	
35–44	5.0	95.0	
45+	5.9	94.1	
**Frequency of face mask wearing inside public spaces**			151.868 (0.000)*
Never	37.8	62.2	
Sometimes	3.0	97.1	
Always	5.0	95.0	
**Frequency of face mask wearing outside public spaces**			196.087 (0.000)*
Never	50.9	49.1	
Sometimes	11.3	88.7	
Always	2.8	97.2	
Total (%)	6.7	93.3	
Total (N)	70	972	

* *p < 0.05*.

## Discussion

Our study demonstrates that majority—more than three quarters of the respondents wore a face mask always outside public spaces and slightly more than half wore a face mask sometimes inside public spaces. This evidence shows that majority of the respondents appreciate the need to wear face masks as a mechanism to prevent COVID-19. However, these findings also reveal that wearing face masks in public places is not universal (25, 26). This is also similar to previous studies that have indicated that a sizeable proportion of the people in low and middle income countries like Nigeria (26), Ethiopia (28), Sudan (27), and Uganda (29) do not actually always wear masks while leaving home to go to public places.

The study also reveals that more than 90% of the respondents believe in the efficacy of wearing face masks in public to prevent transmission or infection by COVID-19. Results suggest that nearly all respondents believe that face masks are effective at preventing COVID-19. These results tend to some extent agree with a recent study in Uganda that shows that a sizeable proportion (68%) of respondents in their study had received information about the use of face masks (29). Our study however, shows a higher percentage of respondents who believe that face masks are effective at preventing COVID-19. This could be because a study by Mboowa and colleagues targeted only high-risk populations in markets, police stations and hospitals while our study targeted all categories of people. However, our results may not follow the same pattern as those from rural areas. This is because remote or rural areas are more likely to experience higher challenges related to meeting the cost of face masks (25), inadequate access to information and education about face mask use (30–32), the perception that people cannot be infected with COVID-19 (33) especially because prevalence of COVID-19 has been reported more in Kampala than other districts (39) and stigma attached to those who wear face masks (36).

Our study shows that almost all respondents who wore the face mask always or sometimes in public spaces believed in the efficacy of wearing a face mask outside public spaces to prevent COVID-19 infection. For example, frequency of face mask wearing (always or sometimes) inside or outside public spaces was significantly related to wearing a face mask outside public spaces. These findings point to the importance of knowledge, access to information about masks and belief in efficacy of face masks as potential determinants of wearing face masks in public spaces to prevent COVID-19 infection (17) particularly among urban dwellers—that were the focus of this study.

## Conclusion

This study reveals that respondents (urban dwellers in Greater Kampala Metropolitan area) believe that face masks are effective at preventing COVID-19. However, this is not yet universal. We understand that change of behavior could take a while, and requires a combination of approaches tailored to different contexts and audience (audience segmentation). A combination of approaches to behavioral change could include mass education (through the use of televisions, radio, newspapers, and posters), interpersonal communication or peer communication, use of interactive digital media (websites, internet newsfeed, social media) and community based approaches (community dialogue and community mobilization). These approaches should be complimented with advocacy campaigns at the political, social, and individual level in order to gain political will, leadership and funds required to effectively engage in sustained behavior change communication. Such approaches can promote continuous face mask wearing as a preventive measure against COVID-19 infection.

## Limitations

Two main limitations emerge from the study. First, the results presented in this study may not be representative because accidental sampling was used to select respondents. Moreover, the study did not map the distribution of respondents in Greater Kampala Metropolitan area. Second, since the study considered only respondents from Greater Kampala Metropolitan area, the results may not be generalizable to other regions of the country.

## Recommendations

The evidence from our study (urban population) that indicates differences in face mask wearing suggests an information gap. Based on the study population (urban population), this study suggests raising awareness about the dangers of COVID-19, infection pathways, and prevention. As Khadka et al. (30) suggest, raising awareness can be in the form of mass education through information sharing, distribution of sanitation materials such as soap, sanitizers as well as face masks. Such strategies can lead to increased use of face masks or even lead to embracing the idea of face mask wearing.

## Data Availability Statement

The original contributions presented in the study are included in the article/supplementary material, further inquiries can be directed to the corresponding author.

## Ethics Statement

The studies involving human participants were reviewed and approved by School of Social Sciences Research Ethics Committee at Makerere University. The patients/participants provided their written informed consent to participate in this study.

## Author Contributions

All authors listed have made a substantial, direct and intellectual contribution to the work, and approved it for publication.

## Conflict of Interest

The authors declare that the research was conducted in the absence of any commercial or financial relationships that could be construed as a potential conflict of interest.

## Publisher's Note

All claims expressed in this article are solely those of the authors and do not necessarily represent those of their affiliated organizations, or those of the publisher, the editors and the reviewers. Any product that may be evaluated in this article, or claim that may be made by its manufacturer, is not guaranteed or endorsed by the publisher.

## Abbreviations

CDC: Centre for Disease Control
KCCA: Kampala Capital City Authority
LICs: Low Income Countries
WHO: World Health Organization.
